# Association between Sleep Duration and Physical Fitness in Children Aged 3–6 Years: A Cross-Sectional Study from China

**DOI:** 10.3390/ijerph19116902

**Published:** 2022-06-04

**Authors:** Xin Xiong, Yinchen Cui, Weinan Zhang, Chenlin Zhao, Jiahui Wu, Haifeng Li, Zhiping Zhen, Jian Sun

**Affiliations:** 1 College of P.E and Sports, Beijing Normal University, Beijing 100875, China; 202031070009@mail.bnu.edu.cn (X.X.); 202121070031@mail.bnu.edu.cn (Y.C.); 202021070016@mail.bnu.edu.cn (W.Z.); 202122070048@mail.bnu.edu.cn (J.W.); 201911070110@mail.bnu.edu.cn (H.L.); 2 Faculty of Athletic Training, Guangzhou Sport University, Guangzhou 510500, China; 3 Nanshan Experimental Education Group, Qilin Middle School, Shenzhen 518051, China; 201821070033@mail.bnu.edu.cn

**Keywords:** children, physical fitness, sleep duration, relevance

## Abstract

Aim: To explore associations between sleep duration and physical fitness (PF) of children aged 3–6 years. Methods: This study investigated the sleep duration and PF data of children aged 3–6 years by stratified random sampling. The restricted cubic spline model and binary logistic regression analysis were mainly used for the empirical analysis of the correlation effect between sleep duration and PF. The final data had a total of 21,857 children, of which 11,245 (51.45%) were boys and 10,612 (48.55%) were girls. Results: The PF level of the children in this study showed a relatively positive level (pass rate = 93.6%), and 19.7% of them had abnormal sleep duration; the results of the restricted cubic spline showed an inverted U-shaped association between the level of PF and the risk of abnormal sleep duration (X^2^ = 28.13, *p* < 0.0001). The results of logistic regression analysis showed that children with abnormal sleep duration were more likely to have a low PF, body morphology and motor ability levels at an OR (95% CI) of 1.077 (1.023–1.133), 1.077 (1.016–1.142) and 1.035 (1.08–1.062), respectively. The results of the bias correlation analysis showed varying degrees of correlation between sleep duration and various components of children’s PF. Conclusion: Insufficient or excessive amounts of sleep were significantly associated with PF in children, with abnormal sleep duration leading to reduced levels of PF and its components.

## 1. Introduction

As the starting stage of life, early childhood is a critical period for the development of good and healthy behaviors, and its physical fitness (PF) status is related to the long-term development of population quality. The 5th National Physical Fitness Monitoring Bulletin of China shows that compared to the 4th monitoring results, the overall level of PF of children aged 3 to 6 years has been improved, with a pass rate of over 90% and a steady trend of change for the positive [1,2]. However, the changes in the components of PF of children vary greatly, such as the increase in obesity prevalence and the decline in strength capacity, flexibility and other motor ability (MA) are the key aspects that will require attention in the promotion of PF of children in the future [3].

The PF of children has a high correlation with factors such as lifestyle and behavioral patterns [4]. With the change in modern lifestyle, the trend of decreasing sleep duration in children has become obvious [5]. Currently, sleep disorders in early childhood have become a global public health concern. Research has shown that the prevalence of sleep disorders such as abnormal sleep behavior disorders, insomnia and delayed sleep phase disorder is about 20–40% and has been confirmed in many countries [6]. Studies in the United States, Australia, Canada and other countries have found that around 30% of children in their countries have sleep disorders [7,8], and studies in China have confirmed that 3- and 4-year-olds are highly susceptible to insufficient sleep duration, and there is a potential trend for the expansion of these sleep disorders [9].

Previous studies have demonstrated the diverse characteristics of sleep effects on PF. A systematic review concluded that there were joint association effects of physical activity, sedentary time and sleep duration on mental health and reported that physical activity and sleep duration were associated with health-related psychological well-being [10]. The relationship between sleep duration and mental health is not a simple linear relationship. A study has confirmed a U-shaped correlation between sleep duration and depression [11]. In addition, some studies have shown that adequate sleep duration is associated with attention, executive function and PF in children [12,13]. Sleep duration and physical activity can have an interactive effect on childhood obesity; meanwhile, appropriate physical activity and sufficient sleep duration can effectively control the prevalence of obesity and maintain PF [14]. It is evident that the health-promoting effects of sleep are reflected in many aspects of mental, physical and life adaptation during childhood.

The promotive effect of sleep on health is an important physiological process that regulates and maintains the health status of the body and is an important indicator of PF [15]. Quality sleep requires adequate sleep duration, regular and appropriate sleep time along with sleep frequency. Some evidence suggests that sleep plays an important role in the promotion of PF in children and adolescents and that good sleep is beneficial for physical and cognitive development, regulation of adverse mental conditions, improvement of healthy behavioral habits and quality of life [16,17]. Poor sleep quality, in contrast, can lead to impaired neurological development and reduced growth hormone secretion, which can have serious negative effects on the healthy development of children in the rapid growth phase. Sleep-related PF problems also have the cross-age effect, with sleep quality problems in 3-year-olds leading to mental and behavioral health problems even at the age of 4 [18].

As sleep is an important factor related to PF in children, few studies have integrated various elements of PF to analyze the correlation between sleep duration and PF in children. Therefore, this study explored the correlation between sleep duration and PF of children by using cross-sectional survey data of children aged 3–6 years in China. It also investigated the relevance of sleep duration to the components of PF, aiming to provide a scientific basis for sleep intervention in the promotion of PF of children.

## 2. Method

### 2.1. Participants

The subjects of the study were from the National Physical Fitness Measurement Standards Manual (Preschool children Version) (NPFMSM) of the General Administration of Sports of China [19]. This project is a national effort to systematically acquire the status of national PF by means of sampling surveys and conducting regular nationwide standardized tests on measurement subjects and analyzing the measurement data in accordance with the national PF measurement indicators promulgated by the state. Since the year 2000, five large-scale nationwide tests have been conducted.

Our cross-sectional survey was conducted between 2018 and 2019 in Guangdong Province, China. In order to obtain representative samples, a total of 26,105 children aged 3 to 6 years were surveyed in this study using stratified random sampling, which distinguished between urban and rural areas. A total of 21,857 subjects (9168 boys and 8856 girls) were included in the study, excluding the data of physical fitness monitoring index test values beyond 3 times standard deviation (M ± 3SD) and the sample of survey index response rate less than 85%. The selection criteria for the participants in this study included: (1) Participants were of appropriate age. All participants were children aged 3–6 years; (2) Absence of disease. Children with illnesses such as contraindications to exercise, cardiovascular disease, neurological or endocrine disorders were not considered; (3) Obtain informed consent from parents or legal guardians. Parents or legal guardians of participants were notified by school teachers the day before the survey and were asked to sign an informed consent form voluntarily. All participants participated in the survey voluntarily. This project was organized by the China Physical Fitness Surveillance Center under the approval of the General Administration of Sports of China.

### 2.2. Measures

This study conducted a survey based on the NPFMSM in terms of both sleep duration and PF. This document was compiled by the General Administration of Sports of China in 2003 [19] and has been applied since then. It has investigated a large amount of data from children aged 3–6 years, which can objectively reflect the real-time status of PF of Chinese children with high reliability and validity.

#### 2.2.1. Demographic Indicators

Demographic indicators include age, gender and residence. We calculated age based on the actual time of completion of the survey.

#### 2.2.2. Sleep Duration

The sleep duration of children consisted of sleep duration at night and daytime nap duration, which were surveyed using the National Physical Fitness Surveillance Questionnaire [20]. The survey included the average time to sleep and wake up within one month. Since the participants were too young to complete the questionnaire independently, the parents and kindergarten teachers reported the sleep duration at night and daytime nap duration, respectively. Based on the duration of evening sleep and lunchtime sleep, the total daily sleep duration of the children was calculated and was divided into abnormal sleep duration and normal sleep duration, which included insufficient sleep duration and excessive sleep duration. The questionnaire was explained by a dedicated person with training experience before filling out the questionnaire.

#### 2.2.3. PF Indicators

The components of PF include indicators related to body morphology (BM) and motor ability (MA). BM indicators consist of height and weight. Height reflects the level of longitudinal bone growth. During the test, the subject stands barefoot and in an upright position on the base of the stadiometer, with the heel, sacrum and both scapulae in contact with the column of the stadiometer, head upright, eyes looking straight ahead and the upper edge of the ear screen level with the lowest point of the lower edge of the orbit, and the test data is read. Body weight reflects the degree of development and nutritional status [21]. The test was performed using a weight scale. During the test, the subject was asked to dress minimally, stand firmly in the center of the scale and read the test data. Recorded data were rounded to 0.1 cm and 0.1 kg.

The MA index consists of six indicators reflecting different athletic abilities, namely, standing long jump reflecting the muscular power of the lower limbs, tennis throwing reflecting upper limb strength, sit-and-reach reflecting flexibility, 10 m shuttle run test reflecting speed ability, balance beam walking reflecting static balance and double-leg timed hop reflecting agility. All tests were completed by professionals with training experience. Each test was conducted three times, and the scores of each test were recorded. The best score was taken for analysis. All participants were verbally encouraged by the instructors during the test to achieve their maximum effort to complete each test. All tests refer to the NPFMSM test requirements for physical health monitoring of young children. The following is the test protocol for each indicator.

SLJ test: The participants stood behind the starting line with their feet together and jumped forward as far as possible. The distance from the starting line to the mat or non-slip surface behind the heel of the point closest to the landing line was measured in meters [19].

TT test: A rectangle of 20 m in length and 6 m in width was drawn on a flat surface, with the end line on one side as the throwing line. During the test, the participants stood behind the throwing line, held the tennis ball with one hand above the head and threw it forward as far as possible. During the throwing process, the feet should not step on or cross the throwing line. The testing results were measured from the throwing line to the first landing point of the ball in meters [19].

SR test: The participants sat on the test mat with legs fully extended and were required to bend their trunks forward and reach the marker with fingertips as far as they could by sliding their hands along the measuring board. The greatest distance contacted by the fingertips past the toes was recorded in centimeters [19].

10-m SRT test: A straight runway of 10 m in length and 1.22 m in width was drawn on the floor. The participants were instructed to step on or cross the line before turning. Participants were required to run as fast as possible from the starting line to the finish line after the signal was given. The stopwatch was stopped when the participants crossed the finish line with one foot, with the results recorded in seconds [19].

BBW test: Participants were required to stand on a balance beam (3 m long, 10 cm wide and 30 cm high) and to walk along the beam as fast as possible from one end to the other with both arms to their sides. If the participant fell off the beam midway, remeasurements were required. The total time was recorded in seconds [19].

DTH test: 10 rectangular soft blocks (10 cm long, 5 cm wide and 5 cm high) were settled every 0.5 m on a flat surface. Participants were required to stand 20 cm behind the first block with both feet together. Participants were required to jump over all the blocks as fast as possible after the start signal was given. Participants were required to redo the test when their foot stepped on or kicked the blocks. The time to complete jumping over all the blocks was recorded in seconds [19].

### 2.3. Data Analysis

According to the scoring standards of the NPFMSM, a five-point scale was used to score each test index of PF. The PF, BM and MA scores were calculated and divided into different evaluation levels, and the criteria for the classification of the levels are shown in Table 1, which considered the levels below the passing level as a low level and the others as a normal level. The standard was promulgated by the China Physical Fitness Surveillance Center in 2003. It is based on the test result of PF indicators for children, and each indicator is graded by the percentile method, which is divided into 5 levels, with scores from 1 to 5 indicating the test scores reflecting the test indicators from the lowest to the highest. The total PF score is obtained by adding up the scores of each indicator. The total score is 40 points, including 10 points for BM and 30 points for MA. It is divided into four grades by percentile method, including excellent, good, pass and fail.

According to the child growth standards published by the World Health Organization, height is used as a reference, and the nutritional status is classified according to the weight for height, which are skinny, thin, normal, overweight and obese [22]. According to the guidelines for sleep hygiene for children aged 0 to 6 published by the National Health and Family Planning Commission of the People’s Republic of China. The sleep duration of young children was categorized as insufficient sleep (<10 h), normal sleep (10–13 h) and excessive sleep (>13 h) [23]. Data from the sleep duration questionnaire and PF monitoring index test data were analyzed using SPSS 26.0. Descriptive statistics and chi-square were used to describe the basic characteristics and distribution differences of PF and sleep trends in children; restricted cubic splines were used to test the relationship between PF and sleep duration dose effect in children; binary logistic regression was used to analyze the effect of sleep duration on the detection rate for low level on PF and its components in children, and the effect was evaluated by OR value and 95% CI was calculated; partial correlation analysis was used to test the correlation between sleep duration and the components of PF, and the test level α = 0.05, two-tailed *p* < 0.05 was considered a statistically significant difference.

## 3. Results

### 3.1. Basic Information and PF Level of Participants

A total of 21,857 children aged 3–6 years were included in this study, of which 11,245 (51.45%) were boys and 10,612 (48.55%) were girls. Meanwhile, it can be seen in Table 2 that 11,245 children were from urban areas and 10,612 children were from rural areas. The average score of PF of the children was 26.14 ± 4.33, which is in the passing grade, and the grade of MB (good) was better than the MA (passing). The average sleep duration just reached the normal range of sleep (10.33 ± 1.12). The distribution of the number of children of different genders in age groups was not statistically significant (*p* > 0.05), while the distribution in different residences (*p* = 0.024) and PF levels (*p* < 0.01) were statistically significant. The overall preference for PF level of children shows a pass rate (pass and above) of 93.6%, with girls (95%) outperforming boys (92.3%), with a decreasing trend of 2.7% with aging. Among the components of PF, BM level was better than MA, as shown in Table 3.

### 3.2. Description of Sleep Duration Characteristics of Children

The prevalence of abnormal sleep duration detected in children was 19.7%, of which 18.4% were sleep deprivation, and 1.3% were excessive sleepiness. Table 4 shows the distribution characteristics of sleep status of children. The chi-square test showed that the differences in the distribution of sleep status of children by gender (X^2^ = 4.069, *p* = 0.131) and nutritional status (X^2^ = 3.294, *p* = 0.864) were not statistically significant, while the differences in the distribution by region (X^2^ = 19.534, *p* < 0.01) and age (X^2^ = 564.679, *p* < 0.01) were statistically significant. The regional differences were reflected in the fact that the sleep quality of rural children was better than that of urban children, the percentage of normal sleep time was more than that of urban children, and the prevalence of sleep deprivation was significantly lower than that of urban children. The age difference is reflected in the overall decrease in sleep duration with aging. The detection rate of sleep deprivation increased with age by 16.8%; both normal sleep and excessive sleep duration decreased with age by 15.2% and 1.6%, respectively.

### 3.3. The Dose–Effect Relationship between Sleep Duration and PF

**Figure 1:**
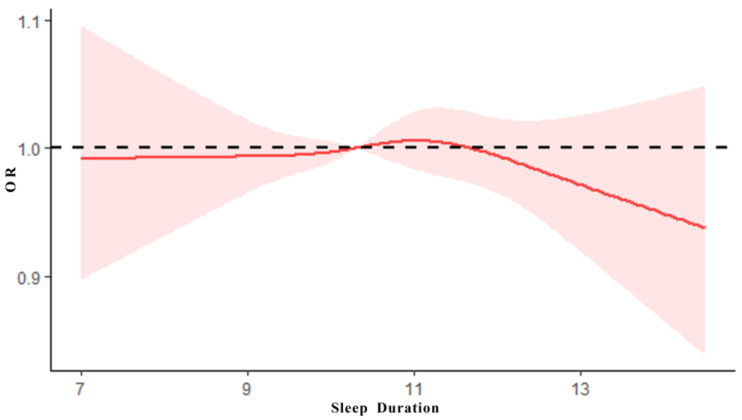
The dose–effect relationship between sleep duration and PF.

After the adjustments for age, gender, family residence and nutritional status, the association between PF and sleep duration was analyzed using a restricted cubic spline model with PF score as the dependent variable. As shown in Figure 1, the results showed that there was an inverted U-shaped association between PF scores and sleep duration in children (X^2^ = 28.13, *p* < 0.0001), and the dose–effect relationship was statistically significant (X^2^ = 9.27, *p* = 0.0097 < 0.01), suggesting that different sleep duration will affect the level of PH, and insufficient sleep and excessive sleep will have a negative impact on PF.

### 3.4. The Association between PF Level and Sleep Duration

**Figure 2:**
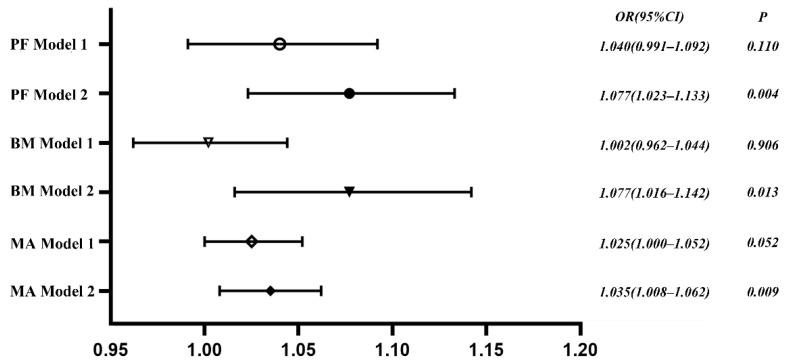
Results of binary logistic regression analysis.

Binary logistic regression analysis was used to explore the correlation between sleep duration and PF levels, with the score of PF, BM and MA as dependent variables (normal = 0, low level = 1). The results showed that without including control variables (Model 1), the association between sleep duration and levels of PF, BM and MA was not statistically significant (*p* > 0.05). After the adjustments for age, gender, home residence and nutritional status (Model 2), the associations of sleep duration with PF, BM and MA levels were statistically significant. Compared with normal sleep duration, the risks of lower levels of PF, BM and MA in sleep abnormalities increase by 1.077, 1.077 and 1.035 times, respectively, as shown in Figure 2.

### 3.5. Correlation of the Components of PF with Sleep Duration

**Figure 3:**
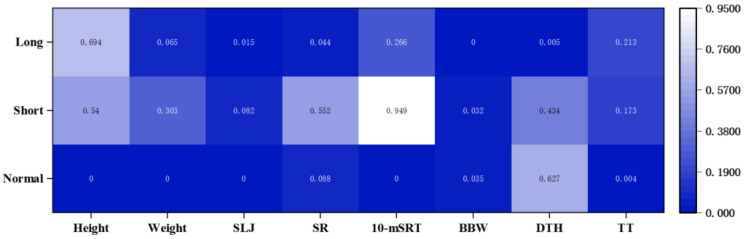
Correlation of the components of PF with sleep duration. SLJ: standing long jump; SR: sit-and-reach; 10-mSRT: 10 m shuttle run test; BBW: balance beam walking; DTH: double-leg timed hop; TT: tennis throwing. Normal: normal sleep duration; Short: insufficient sleep duration; Long: excessive sleep duration. The numbers in the table indicate significance coefficient, and the significance level is *p* < 0.05.

So as to explore the core factors of the effect of sleep duration on PF in children, the correlations between the components of PF and sleep duration were examined using partial correlation analysis, which controlled for age, gender, family residence and nutritional status. The results showed that most of the components of PF in the normal sleep group were statistically significantly correlated with sleep duration (*p* < 0.01). Among them, sleep duration was significantly negatively correlated with morphological, strength capacity and balance ability (R_Height_ = −0.138, R_Weight_ = −0.123, R_SLJ_ = −0.043, R_TT_ = −0.022, R_B__BW_ = −0.004, *p* < 0.05), significantly positively correlated with speed ability (R_10-mSRT_ = 0.039, *p* < 0.01), while the correlation with flexibility and agility was not statistically significant. It indicates that increasing the sleep duration during normal sleep time decreases the performance of the above-mentioned motor abilities, while the performance of speed ability requires more sleep time to achieve superior performance. Only the correlation between balance ability and sleep duration was statistically significant in the sleep-deprived group (*p* = 0.032 < 0.05). The excessive sleepiness group, on the other hand, had a statistically significant correlation between upper body strength, flexibility, balance and agility along with sleep duration (*p* < 0.05), as shown in Figure 3.

## 4. Discussion

The level of PF is a critical component in evaluating health [24], and this study analyzed the correlation between sleep duration and PF through a large-scale survey of cross-sectional data from children aged 3 to 6 years. It was found that there is a strong association between sleep duration and PF in children. Both insufficient and excessive sleep duration increase the detection rate of low PF levels compared to normal sleep duration, suggesting that scientific sleep scheduling may be a protective factor for PF promotion in children.

This study completes data collection based on the NPFMSM, which aims to understand the current situation and change patterns of national PF in China, enrich and improve the national PF and health surveillance system and database, and serve to improve the level of scientific fitness guidance and public service capacity of national fitness and improve the PF and health level of all nationals. The results of this study show that the overall level of PF of children is relatively positive, with more than 90% of children reaching a passing grade and girls being superior to boys. Among the components of PF, the level of BM is better than MA, which is basically consistent with the results of “the fifth national PF monitoring in China” [1]. The results of this study differed from the results of regional studies on the PF of children in China [25], and the PF level of participants in this study was higher than most other existing studies [26,27,28].

Sleep deprivation is the main manifestation of sleep problems in children [29]. The detection rate of abnormal sleep duration among participants in this study was 19.7% (18.4% for sleep deprivation and 1.3% for excessive sleepiness), which was significantly lower than the sleep duration survey of urban preschool children in China [29]. There were significant differences in the distribution of sleep duration by residence and age, with rural children having more adequate sleep duration. In addition, children’s sleep duration has an increasing age-standardized trend, and the detection rate of insufficient sleep duration gradually increases with aging. The trend in the distribution of sleep duration in children is consistent with the results of most studies, suggesting that aging may be one of the important reasons for the decrease in sleep duration in children [30,31].

This study found an inverted U-shaped association between sleep duration and PF levels in children, with both insufficient sleep and excessive sleep duration increasing the risk of developing low PF levels; this association occurred not only in children, but similar results were found in studies of the association between sleep and health in adults [31,32]. This study suggests that excessive sleep duration had a greater negative effect on PF of children, which is statistically significant, and that the trend of the effect increased with sleep duration. Moreover, the negative effect of insufficient sleep duration on the PF of children was relatively stable. It can be argued that in the management of sleep duration in children, we should pay more attention to avoiding excessive sleep duration. A study showed that because of the high correlation of sleep on health-related factors such as psychological behavior, cognitive function and cardiovascular function in adolescents, studies had been proposed to include sleep in the evaluation system of children’s development and PF [33]. A logistic regression analysis revealed that the endogeneity of the regression model was reduced by adjusting for control variables. The results showed that the regression model of sleep duration and PF and its components in children were statistically significant. Sleep abnormalities significantly increased the risk of developing low PF in children. We have adjusted the effects of gender, age and nutritional status on PF in children in this study. We found that there was an independent association effect between sleep and PF, with the risk of low PF in children with abnormal sleep being 1.077 times greater than normal sleep duration. A UK survey investigated 108 preschool children aged 3–5 years, and it found that children with persistent sleep deprivation had increased symptoms of attention deficit, hyperactivity disorder, depression and anxiety at 3 years compared to controls. In addition, children with these symptoms typically had lower levels of PF [34]. However, the mechanism of the association between sleep duration and PF in young children is not completely clear, and the correlated impact is reflected in the joint effect of sleep duration on the components of PF.

In terms of BM, existing studies have shown that sleep plays an important role in the physical development of children [35]. Weight and height are typical indicators of BM, and both insufficient sleep and excessive sleep duration increase the risk of obesity in children [36], which is consistent with the results of our study. The effect of sleep on weight is mainly due to obesity caused by insufficient sleep duration, which may be related to energy imbalance [37]. There is insufficient evidence to confirm the dose effect of sleep duration and height development, but most studies suggest that adequate sleep duration is a protective factor to ensure normal height development in childhood [38].

Regarding MA, normal sleep duration had a strong correlation with the motor performance of children. MA is the ability of the body’s neuromuscular system to overcome resistance, and this study found that MA in the normal sleep group decreased with increasing sleep duration. Some studies confirm that insufficient sleep duration significantly decreases grip strength [39,40]. The reason for the decrease in strength due to sleep is not yet clear, but some studies have confirmed that the insufficient sleep group is more prone to sarcopenia, as well as symptoms such as decreased muscle mass, compared to the group with normal sleep [41]. Speed is the body’s ability to move quickly, and adequate sleep duration can facilitate its performance [42]. In addition, sleep duration in children also shows varying degrees of correlation with balance, flexibility and agility. Some studies have argued the mechanism of the effect of sleep duration on MA from an energy metabolism perspective. The main reason for the effect of sleep on MA was to promote the recovery of nervous system function by decreasing energy expenditure [43]. At the same time, sleep promotes protein synthesis and mobilizes free fatty acids in the blood to provide energy for MA from an energy metabolism perspective [44]. Other researchers have shortened the sleep duration of participants in sleep intervention experiments, and these subjects showed increased subjective fatigue and decreased exercise performance [45].

According to the evaluation index system of NPFMSM, children’s PF is reflected through eight indexes in two dimensions of BM and MA. This study found that different sleep duration has different relationships with the components of PF. Abnormal sleep duration will increase the risk of low PF; at the same time, the association between normal sleep duration and the components of PF is inconsistent, indicating that sleep duration is a risk factor leading to the decline of PF. In the future, it is necessary to research further the potential biological mechanism between sleep duration with different PF factors and explore the optimum value of sleep duration for the normal growth and development of children’s PF factors.

### Limitation and Implication

There are some limitations of this study. This study is a cross-sectional study, and no causal inference can be made about sleep duration and PF of children. All the survey data on sleep duration were completed by parents and teachers, which were not objective measurements, and the statistical results may be biased due to measurement errors. Further consideration should be given to the association between other factors of sleep and PF, including sleep disorders and sleep quality, to optimize the association between sleep and PF.

## 5. Conclusions

This study analyzed the association between sleep duration and PF of children aged 3–6 years through cross-sectional survey data of a large population sample, which showed that sleep duration had a strong correlation with both PF and its constituent factors, suggesting that the development of PF can be effectively promoted by improving sleep duration of children.

## Figures and Tables

**Table 1:** Classification of PF evaluation level for children.

Level	Standard	Items		
		PF Score	BM Score	MA Score
L1 (Excellent)	[77.6–100%]	32–40	9–10	24–30
L2 (Good)	[67.6–77.5%]	28–31	7–8	21–23
L3 (Passing)	[47.6–67.5%]	20–27	5–6	15–20
L4 (Fail)	[0–47.5%]	0–19	0–4	0–14

PF: physical fitness; BM: body morphology; MA: motor ability.

**Table 2:** Demographic and clinical baseline of the participants.

	Number	Age	Residence (N)		PF Status			Sleep Duration (Hour)
			Urban	Rural	PF Score	MB Score	MA Score	
Boys	11,245	4.46 ± 1.04	6251	5738	25.86 ± 4.44	7.23 ± 1.89	18.61 ± 3.74	10.33 ± 1.12
Girls	10,612	4.44 ± 1.04	4994	4874	26.44 ± 4.17	7.46 ± 1.72	18.99 ± 3.55	10.34 ± 1.13
Total	21,857	4.45 ± 1.04	11,245	10,612	26.14 ± 4.33	7.34 ± 1.81	18.80 ± 3.65	10.33 ± 1.12

**Table 3:** Characterization of PF level of children.

Characteristic		Total [*n* = 21,857, Case (%)]	Boys [*n* = 11,245, Case (%)]	Girls [*n* = 10,612, Case (%)]	X^2^	*p*
Age					4.037	0.258
3		4979 (22.8)	2507 (22.4)	2472 (23.3)		
4		6263 (28.6)	3220 (28.6)	3043 (28.7)		
5		6445 (29.5)	3334 (29.6)	3111 (29.3)		
6		4170 (19.1)	2184 (19.4)	1986 (18.7)		
Residence					5.082	0.024
	Urban	11,989 (54.9)	6251 (55.6)	5738 (54.1)		
	Rural	9868 (45.1)	4994 (44.4)	4874 (45.9)		
PF level					86.561	<0.01
	L1	2344 (10.7)	1125 (10.0)	1219 (11.5)		
	L2	6030 (27.6)	2988 (26.6)	3042 (28.7)		
	L3	12,087 (55.3)	6262 (55.7)	5825 (54.8)		
	L4	1396 (6.4)	870 (7.7)	526 (5)		
BM level					126.773	<0.01
	L1	6497 (29.7)	3336 (29.7)	3161 (29.8)		
	L2	9261 (42.3)	4471 (39.8)	4790 (45.1)		
	L3	4077 (18.7)	2197 (19.5)	1880 (17.7)		
	L4	2022 (9.3)	1241 (11.0)	781 (7.4)		
MA level				56.431		<0.01
	L1	2168 (9.9)	1048 (9.3)	1120 (10.6)		
	L2	5025 (23.0)	2530 (22.5)	2495 (23.5)		
	L3	11,982 (54.8)	6114 (54.4)	5868 (55.3)		
	L4	2682 (12.3)	1553 (13.8)	1129 (10.6)		

**Table 4:** Differences in the distribution of children’s sleep duration.

Characteristic		Insufficient (<9 h)	Normal (9–13 h)	Excessive (>13 h)	X^2^	*p*
Gender					4.069	0.131
	Boys (11,245)	2038 (18.1)	9051 (80.5)	156 (1.4)		
	Girls (10,612)	1976 (18.6)	8518 (80.3)	118 (1.1)		
Residence					19.534	<0.01
	Rural (9868)	1721 (17.4)	7997 (81.0)	150 (1.5)		
	Urban (11,989)	2293 (19.1)	9572 (79.8)	124 (1.0)		
Age					564.679	<0.01
	3 (4979)	526 (10.6)	4359 (87.5)	94 (1.9)		
	4 (6263)	962 (15.4)	5186 (82.8)	115 (1.8)		
	5 (6445)	1382 (21.4)	5011 (77.8)	52 (0.8)		
	6 (4170)	1144 (27.4)	3013 (72.3)	13 (0.3)		
Nutritional status					3.924	0.864
	Skinny	241 (19.6)	972 (79)	17 (1.4)		
	Thin	455 (18.9)	1915 (79.5)	32 (1.3)		
	Normal	2657 (18.2)	11,795 (80.6)	185 (1.3)		
	Overweight	243 (17.7)	1113 (81.1)	16 (1.2)		
	Obese	418 (18.9)	1774 (80.1)	24 (1.1)		
Total (21,857)		4014(18.4)	17,569(80.4)	274(1.3)		

## Data Availability

To protect patient privacy, study data are available on request to the corresponding author.
